# Association of childhood health and socioeconomic status with dementia risk in older age: a cross-sectional study using the Indonesia Family Life Survey 2014–2015

**DOI:** 10.1136/bmjopen-2024-093896

**Published:** 2025-08-16

**Authors:** Tung Le, Amanda Lee, James Gilleen, Asri Maharani

**Affiliations:** 1 The University of Manchester, Manchester, UK; 2 Manchester Metropolitan University, Manchester, UK

**Keywords:** Cross-Sectional Studies, Aging, Dementia

## Abstract

**Abstract:**

**Objectives:**

This study aims to investigate the associations between childhood health, childhood socioeconomic status and dementia risk in later life, and to assess the potential modifying effects of their interaction. The study also accounted for key confounders to better clarify these relationships within the Indonesian population.

**Design:**

Cross-sectional study.

**Setting:**

Indonesia.

**Participants:**

6693 aged 50+.

**Results:**

Individuals in the ‘unhealthy’ childhood health cluster had 1.17 times higher odds of dementia risk compared with the ‘healthy’ cluster (95% CI: 1.00 to 1.38), a borderline association, while those in the ‘poor socioeconomic status’ cluster had 1.39 times higher odds compared with the ‘non-poor’ cluster (95% CI: 1.15 to 1.68). No significant interaction was found between childhood health and socioeconomic status on either the multiplicative (OR=0.88, 95% CI: 0.30 to 2.57) or additive scale (all relative excess risk due to interaction, attributable proportion and synergy index measures non-significant). Older age, lower education, lower wealth, lower social capital and higher depression scores are significantly associated with increased dementia risk.

**Conclusion:**

This study finds that both childhood health and socioeconomic status independently influence dementia risk in later life. No significant interaction between these two early-life factors was found, suggesting that their effects on dementia risk operate independently rather than synergistically. Using nationally representative Indonesian data, the findings highlight the importance of addressing early-life adversity in dementia prevention and call for standardised definitions to improve research comparability, particularly in low-income and middle-income countries contexts.

**Strengths and limitations of this study**

- This study uses data from the Indonesia Family Life Survey, a large and nationally representative dataset.
- Childhood health and socioeconomic status were classified using latent class analysis to reduce recall bias and measurement error.
- Multiple imputation using chained equations (MICE) was applied to handle missing data under the assumption that the data were missing at random.
- Dementia risk was inferred from a brief cognitive screening tool (TICS) rather than clinical diagnosis, which may not fully capture the complexity of dementia risk.
- The cross-sectional study design limits causal inference and is susceptible to survival and recall biases.

## Introduction

 Dementia is a major global public health concern because it progressively worsens over time and currently has no cure.^1^ It is a chronic and progressive syndrome characterised by deterioration in memory, thinking, behaviour and the ability to perform everyday activities.^2^ Beyond its impact on individuals, dementia imposes substantial emotional and socioeconomic burdens on families and healthcare systems, highlighting the need to identify modifiable risk factors across the life course.^3^

Growing evidence suggests that early-life conditions play a crucial role in shaping dementia risk in later life.^4^,^6^ This relationship appears consistent across diverse geographical and economic contexts, although specific risk patterns vary. The association between childhood health, childhood socioeconomic status (SES) and later-life dementia risk has been particularly well-documented in low-income and middle-income countries (LMICs) settings. In Malaysia, Momtaz *et al* found that childhood food insufficiency, measured through recurrent experiences of hunger, predicted an 81% higher risk of dementia in older adulthood.^7^ Complementary findings from China demonstrate that favourable childhood SES, such as higher parental education and greater household financial stability, are associated with lower midlife dementia risk.^8^

Studies from high-income countries present both confirmatory and contradictory evidence. A US-based study demonstrated that individuals whose parents attained higher levels of education tend to exhibit significantly better cognitive performance in later life.^9^ Similarly, a Japanese longitudinal study found that individuals who experienced high levels of parental involvement in childhood scored approximately 3.7 points higher on the Quick Mild Cognitive Impairment test in later life compared with those with low involvement.^10^ Tsang *et al*’s multicentre analysis of UK and US cohorts further established that childhood financial hardship and parental occupational status predicted accelerated dementia risk trajectories.^11^ However, an Australian longitudinal study found no association between adverse childhood experiences and late-life dementia risk,^12^ while an Irish study paradoxically observed improved cognitive functioning with greater childhood infectious disease burden.^13^

Hence, critical gaps remain in the literature. First, the relationship between childhood health, SES and dementia risk remains controversial, with conflicting results across studies. Second, most existing research examines childhood health and SES separately, neglecting potential interactions between the two. This study aims to address these gaps by investigating the combined influence of childhood health and SES on dementia risk in Indonesia, a setting where such evidence remains scarce.

We hypothesise that poor childhood health and lower SES significantly increase the likelihood of dementia in later life. Additionally, this study accounts for potential confounding factors, including age, gender, marital status, social connections, physical health, behavioural risks and depression. By elucidating these associations, our findings may inform targeted interventions and policies to mitigate dementia risk from a life-course perspective. Given the growing dementia burden in LMICs, this research could also provide a framework for similar studies in other resource-limited settings, ultimately supporting global efforts to address dementia’s escalating impact.

## Methods

### Study design and participants

This cross-sectional study used anonymous, public data from Wave 5 (2014–2015) of the Indonesia Family Life Survey (IFLS), a nationally representative longitudinal survey.^14^ The IFLS collects comprehensive socioeconomic and health information through face-to-face interviews and is part of the Health and Retirement Study (HRS) family of harmonised ageing surveys conducted across multiple countries.^15^ Wave 5 was selected for this study because it was the first to include detailed retrospective self-reported information on childhood health and SES, essential for our research objectives. From the original Wave 5 sample (n=36 391 participants aged ≥15 years across 13 provinces), we restricted our analysis to adults aged ≥50 years (n=27 909 excluded) with complete childhood health and SES data (n=1789 excluded), resulting in a final analytical sample of 6693 participants. This age cut-off ensured capture of the target population at elevated dementia risk while maintaining a sufficient sample size for robust analysis.^11 16^

### Childhood health measures

We assessed childhood health using five indicators: general childhood health, school absence due to health issues, bed confinement, hospitalisation and childhood hunger. General health was self-rated by respondents on a scale from 1 (Excellent) to 5 (Poor), then categorised into two groups: 1–3 (Good and better) and 4–5 (Fair and poor). The other four indicators were measured via yes/no questions: “Did you miss school for a month or more due to health issues?”, “Were you confined to bed for a month or more due to health reasons?”, “Were you hospitalised for a month or more for health reasons?” and “Did you experience hunger during childhood?”. These childhood health variables were then included in latent class analysis (LCA) to identify clusters of childhood health.

### Childhood SES measures

Childhood SES was assessed using five indicators: overcrowding, availability of electricity, availability of running water, availability of indoor toilets and number of books in the household. Overcrowding was determined by dividing the number of rooms (excluding kitchen, bathrooms and hallways) by the number of people in the household when the participant was 12. A ratio of less than one indicated overcrowding.^17^ Electricity availability was measured with a yes/no question: “When you were 12, did your household have electricity?” Running water was assessed by a yes/no question: “When you were 12, what was the main water source for drinking in your household?” Responses of “Piped water” or “Well/pump water” indicated water availability, while any other responses did not. Indoor toilet availability was evaluated with: “When you were 12, where did the majority of household members go to the toilet?”. Options “Own toilet with septic tank” and “Own toilet without septic tank” indicate the availability of an indoor toilet. Number of books was assessed by: “Approximately how many books were there in your home when you were 12?” Responses were categorised as: “None or very few (0–10 books),” “Enough to fill one shelf (11–25 books),” “Enough to fill one bookcase (26–100 books),” “Enough to fill two bookcases (101–200 books),” and “Enough to fill two or more bookcases (more than 200 books).” Responses were used to create two groups: “Very few” indicated fewer than 10 books and “All other responses” indicated 10 or more books. These childhood SES variables were then added to the LCA to identify clusters.

### Dementia measures

Dementia was classified using the cognitive function score from the Telephone Interview for Cognitive Status (TICS) scale, a standardised tool used in large epidemiological studies.^18^ It has been widely used in ageing research across both high-income and LMICs.^19^,^21^ In the IFLS, TICS was administered through face-to-face interviews.^22^ The cognitive assessment comprised three tests: (1) an episodic memory test, in which participants recall as many of 10 words as possible immediately after administration, and again at the end of the session (delayed recall) (maximum score: 20 points); (2) a serial subtraction test, in which participants verbally subtract 7 from 100 five times (maximum score: 5 points); and (3) a backward counting test, in which participants count backwards from 20 for 10 numbers (maximum score: 2 points). The total score ranged from 0 to 27 points, with higher scores indicating better cognitive performance. Following the classification approach developed by Langa *et al* for the HRS and its international sister studies,^23^ participants were categorised into three groups based on their total TICS-27 score: 12–27 points indicating normal cognitive function, 7–11 points representing cognitively impaired but not demented (CIND) and 0–6 points signifying a high risk of dementia. These cut-offs have been used in prior studies and shown to have acceptable sensitivity and specificity in distinguishing levels of cognitive function in population-based settings where clinical diagnosis is not available.^23 24^ While a recent study using IFLS data applied the same categorisation to assess cognitive outcomes,^25^ these thresholds lack formal validation in Indonesia, so prevalence estimates should be interpreted cautiously.

### Covariates

This study included several covariates: age (continuous), sex (male as reference), marital status (single, married, divorced and widowed, with widowed as reference), education level (primary and lower, secondary, and college or above, with college or above as reference) and employment status (employed as reference). Per capita household expenditure (PCE), reflecting households’ ability to meet needs and living standards in Indonesia, was calculated by dividing total household expenditure by the number of household members.^26^ PCE was used as a proxy for personal income and wealth, categorised into quintiles (first quintile as poorest, fifth quintile as richest, with the fifth quintile as reference). Social capital was measured as the total number of activities within 1 year (continuous). Smoking status was categorised as current smoker, ex-smoker or non-smoker (non-smoker as reference). The total number of chronic diseases (continuous) included heart disease, hypertension, stroke, cancer, chronic lung disease and diabetes. Finally, depression was assessed using a continuous score from the Center of Epidemiologic Studies Depression Scale, 10-item version(CES-D 10), with a lower score indicating lower depression.

### Statistical analysis

Most studies examining the influence of childhood conditions on dementia in later life have used retrospectively collected childhood conditions as the essential exposure.^27 28^ However, the use of such retrospective information gives rise to the potential problem of recall bias, especially among respondents with dementia risk. To address this bias, our study used LCA to objectively group childhood health and SES, reducing reliance on self-reports (see online supplemental figure S1).

First, LCA was performed on childhood health and SES variables separately to identify clusters. The number of distinct clusters identified and chosen for final analyses was dependent on various criteria: a lower Akaike’s Information Criteria (AIC) and Bayesian Information Criteria (BIC) with each class added to the model, an entropy value of at least 0.8 (indicating strong model accuracy and reliability) and at least 5% of the respondents within each class.^29^ Second, the frequency and percentage of each trait within the identified clusters were calculated. Covariates were reported by subgroup, with categorical variables presented as frequencies and percentages, and continuous variables as means and SD. We performed bivariate analysis using the χ² test for categorical variables and the Kruskal-Wallis test for continuous variables. Then, multivariate ordinal logistic regression analyses were used to estimate the associations between childhood health, childhood SES and their interaction with dementia risk within the identified latent classes. Three models were applied: Model 1 adjusted for age and sex; Model 2 further adjusted for SES and lifestyle factors that is, employment, marital status, education level, wealth quintile and social activities; and Model 3 additionally added health behaviours and health status that is, smoking status, number of chronic diseases and depression scores. Finally, we assessed multiplicative and additive interactions between childhood health and childhood SES on dementia risk, following guidelines for interaction analysis.^30^

### Sensitivity analysis

To assess the robustness of the main findings, we conducted a sensitivity analysis using multiple imputation using chained equations (MICE) to address missing data, following White and colleagues.^31^ The multivariate ordinal logistic regression models were re-estimated using the imputed data and compared with the original results to examine whether the observed associations remained consistent.

### Patient and public involvement

Patients and the public were not involved in the design, conduct, reporting or dissemination plans of this research.

## Results

Descriptive characteristics of the analytic sample (n=6693), along with the extent of missing data, are presented in online supplemental table S2. Based on the categorisation of the TICS score, 47.29% of participants (n=2747) were classified as cognitively normal, 37.05% (n=2152) as having CIND and 15.67% (n=910) as having a high risk of dementia. Missing data for TICS scores, education and wealth quintile were 13.21%, 0.18% and 7.34%, respectively, while all other variables had complete data.

The results of the LCA for the childhood health variable group identified two models. Based on the model fit (see online supplemental table S3), the 2-class model was chosen as the 3-class model included a cluster that constituted only 0.33% of the sample. Similarly, analysis of the childhood SES variable group also produced two models. We selected the 2-class model due to its lower BIC index than the 3-class model. The chosen models for childhood health and SES demonstrated high entropy indexes, 0.95 and 0.96, respectively, indicating strong model accuracy and reliability.

Online supplemental table S4 presents the characteristics of variables observed between the two latent classes of childhood health and SES. The ‘healthy’ and ‘unhealthy’ clusters comprised 6083 (90.89%) and 610 (9.11%) individuals, respectively. The ‘healthy’ cluster exhibited superior health outcomes, with fewer instances of school absences, less time bedridden due to health issues, fewer hospitalisations and lower incidences of hunger compared with the ‘unhealthy’ cluster. The result from LCA for childhood SES is presented in online supplemental table S5. The ‘non-poor SES’ and ‘poor SES’ clusters comprised 762 (11.39%) and 5931 (88.61%) individuals, respectively. The ‘poor SES’ cluster experienced higher levels of overcrowding, lack of basic amenities such as electricity, water and toilets, and fewer individuals owning more than 10 books compared with the ‘non-poor SES’ cluster.

Table 1 highlights gender, education, SES and health disparities in the analytic sample. Among participants, women are more likely to experience ‘unhealthy’ childhoods (51.12%; p=0.012) and belong to the ‘poor SES’ cluster (43.44%; p=0.039). Educational attainment differed by childhood health and SES: 75.25% of those with ‘unhealthy’ childhoods have only primary education or less, compared with 67.69% of those with ‘healthy’ childhoods (p<0.001). Similarly, the ‘poor SES’ childhood cluster has lower education levels than the non-poor (p<0.001). Smoking prevalence is higher among those with ‘unhealthy’ childhoods (36.78%) than ‘healthy’ childhoods (31.78%, p=0.001) and among the ‘poor SES’ cluster (32.96%) than the ‘non-poor SES’ (27.82%, p=0.015). The poorest quintile has a higher prevalence of ‘poor SES’ childhoods than the non-poor cluster (23.27% vs 9.75%, p<0.001). Unemployment is more common among those with ‘unhealthy’ childhoods (27.93%) than ‘healthy’ childhoods (32.47%, p=0.01). Conversely, the ‘non-poor SES’ childhood cluster has a higher proportion of people not working (39.24%) than the ‘poor SES’ childhood cluster (30.99%, p<0.001), possibly due to the older age of the study population retiring after achieving economic stability. Depression scores are higher among those with ‘unhealthy’ childhoods (16.87) and ‘poor SES’ childhoods (15.72).

**Table 1:** Descriptive characteristics of participants by childhood health and childhood SES, showing mean (SD), frequency (%) and significance values of Kruskal-Wallis and χ² tests

Characteristics	Healthy N=5891	Unhealthy N=802	P value	Non-poor SES N=762	Poor SES N=5931	P value
Age, mean (SD)	59.95±8.14	59.36±7.78	0.0832	57.40±6.87	60.20±8.19	0.001
Sex, frequency (%)			0.012			0.039
Female	2733 (46.39)	410 (51.12)		331 (43.44)	2812 (47.41)	
Male	3158 (53.61)	392 (48.88)		431 (56.56)	3119 (52.59)	
Marital status, frequency (%)			0.218			<0.001
Single	61 (1.04)	9 (1.12)		19 (2.49)	51 (0.86)	
Married	4391 (74.54)	617 (76.93)		577 (75.72)	4431 (74.71)	
Separated	216 (3.67)	34 (4.24)		30 (3.94)	220 (3.71)	
Widower	1223 (20.76)	142 (17.71)		136 (17.85)	1229 (20.72)	
Education, frequency (%)			<0.001			<0.001
Primary and lower	3981 (67.69)	602 (75.25)		209 (27.50)	4374 (73.87)	
Secondary	1421 (24.16)	151 (18.88)		371 (48.82)	1201 (20.28)	
College and higher	479 (8.14)	47 (5.88)		180 (23.68)	346 (5.84)	
Employment, frequency (%)			0.01			<0.001
No	1913 (32.47)	224 (27.93)		299 (39.24)	1838 (30.99)	
Yes	3978 (67.53)	578 (72.07)		463 (60.76)	4093 (69.01)	
Wealth quintile, frequency (%)			0.229			<0.001
Poorest	1171 (21.47)	182 (24.33)		65 (9.75)	1288 (23.27)	
Poor	1066 (19.55)	144 (19.25)		87 (13.04)	1123 (20.29)	
Average	1014 (18.59)	148 (19.79)		102 (15.29)	1060 (19.15)	
Rich	1078 (19.77)	139 (18.58)		137 (20.54)	1080 (19.51)	
Richest	1125 (20.63)	135 (18.05)		276 (41.38)	984 (17.78)	
Social capital, mean (SD)	2.22±1.95	2.26±2.01	0.9079	2.39±2.15	2.21±1.93	<0.001
Smoking, frequency (%)			0.001			0.015
Smoker	1872 (31.78)	295 (36.78)		212 (27.82)	1955 (32.96)	
Past smoker	546 (9.27)	88 (10.97)		73 (9.58)	561 (9.46)	
Non-smoker	3473 (58.95)	419 (52.24)		477 (62.60)	3415 (57.58)	
Chronic diseases, mean (SD)	0.41±0.66	0.45±0.68	0.1154	0.51±0.73	0.40±0.65	<0.001
Depression, mean (SD)	15.48±4.57	16.87±5.29	0.001	15.07±4.25	15.72±4.73	<0.001

SES, socioeconomic status.

To isolate net associations with high risk of dementia, we built three regression models, and the findings are shown below (table 2).

**Table 2:** Multivariable logistic regression results showing the association between childhood health and dementia risk outcome, adjusted for covariates

	Model 1 OR (95% CI)	Model 2 OR (95% CI)	Model 3 OR (95% CI)
Clusters			
Healthy	Reference	Reference	Reference
Unhealthy	1.30 (1.12 to 1.52)	1.21 (1.03 to 1.42)	1.17 (1.00 to 1.38)
Age	1.06 (1.05 to 1.06)	1.05 (1.04 to 1.06)	1.05 (1.04 to 1.06)
Sex			
Female	0.83 (0.75 to 0.91)	1.04 (0.92 to 1.17)	0.96 (0.81 to 1.14)
Male	Reference	Reference	Reference
Employed			
Yes		Reference	Reference
No		1.02 (0.90 to 1.15)	1.03 (0.91 to 1.17)
Marital status			
Single		1.39 (0.79 to 2.44)	1.39 (0.79 to 2.44)
Married		0.96 (0.82 to 1.11)	0.96 (0.83 to 1.12)
Separated		1.14 (0.84 to 1.55)	1.12 (0.82 to 1.52)
Widower		Reference	Reference
Education			
Primary and lower		6.46 (4.97 to 8.38)	6.04 (4.64 to 7.86)
Secondary		2.10 (1.61 to 2.75)	2.01 (1.54 to 2.64)
College and higher		Reference	Reference
Wealth quintile			
Poorest		1.53 (1.28 to 1.82)	1.51 (1.27 to 1.80)
Poor		1.51 (1.27 to 1.80)	1.50 (1.26 to 1.79)
Average		1.17 (0.98 to 1.40)	1.17 (0.98 to 1.40)
Rich		1.16 (0.98 to 1.38)	1.17 (0.98 to 1.39)
Richest		Reference	Reference
Social capital		0.95 (0.92 to 0.98)	0.95 (0.92 to 0.98)
Smoking			
Smoker			1.11 (0.94 to 1.31)
Past smoker			1.07 (0.85 to 1.33)
Non-smoker			Reference
Chronic diseases			0.94 (0.87 to 1.03)
Depression			1.03 (1.01 to 1.04)

In Model 1, after controlling for age and gender, the ‘unhealthy’ childhood cluster had a 1.30 times higher dementia risk than the ‘healthy’ one (95% CI: 1.12 to 1.52). This risk was reduced to 1.21 times higher in Model 2, which included employment, marital status, education, wealth quintile and social capital (95% CI: 1.03 to 1.42). In the fully adjusted model including depression, the association between childhood health and dementia risk was no longer statistically significant, suggesting that depression may mediate this relationship. All models consistently identified older age as a substantial risk factor for dementia, with each additional year raising the risk by 5%–6%. Interestingly, while women had a lower risk compared with men in Model 1 (OR=0.83, 95% CI: 0.75 to 0.91), this difference was not significant when other covariates were included. Education consistently played an important role; individuals with only primary or lower education had a high risk of dementia 6.04 times greater than those with a college education (95% CI: 4.64 to 7.86), while those with secondary education had a risk 2.01 times higher (95% CI: 1.54 to 2.64) in the final model. Wealth also had a substantial impact, with individuals in the poorest wealth quintile facing a risk 1.51 times greater than those in the wealthiest quintile. Attending more social events had a modest protective impact, lowering the risk by 5% in Models 2 and 3. Depression elevated the dementia risk by 3% for each score increase. Other characteristics included employment, marital status, smoking and chronic conditions, which conferred no significant risk of having dementia.

Table 3 shows regression results across three models that are consistent with those found relating to childhood health in table 2. The findings also demonstrate a broad consistency in the relationships between covariates and dementia risk. However, there is a significant difference between the ‘poor SES’ childhood cluster and the ‘non-poor SES’ cluster in terms of dementia risk. In Model 1, the dementia risk for the ‘poor SES’ childhood cluster was 2.53 times larger than that for the ‘non-poor SES’ cluster (95% CI: 2.14 to 2.98). This risk was lowered to 1.39 times higher in Model 2 (95% CI: 1.15 to 1.69) and maintained at 1.39 times higher in the final model (95% CI: 1.15 to 1.68). Overall, the results from this table reinforce the robustness of the associations between covariates and the risk of dementia relating to childhood health (table 2).

**Table 3:** Multivariable logistic regression results showing the association between childhood SES and dementia risk outcome, adjusted for covariates

	Model 1 OR (95% CI)	Model 2 OR (95% CI)	Model 3 OR (95% CI)
Clusters			
Non-poor SES	Reference	Reference	Reference
Poor SES	2.53 (2.14 to 2.98)	1.39 (1.15 to 1.69)	1.39 (1.15 to 1.68)
Age (years)	1.05 (1.05 to 1.06)	1.05 (1.04 to 1.05)	1.05 (1.04 to 1.06)
Sex			
Female	0.81 (0.73 to 0.90)	1.03 (0.91 to 1.16)	0.95 (0.81 to 1.13)
Male	Reference	Reference	Reference
Employed			
Yes		Reference	Reference
No		1.03 (0.91 to 1.17)	1.04 (0.92 to 1.18)
Marital status			
Single		1.44 (0.82 to 2.53)	1.44 (0.82 to 2.53)
Married		0.95 (0.82 to 1.10)	0.96 (0.83 to 1.11)
Separated		1.14 (0.84 to 1.55)	1.12 (0.82 to 1.52)
Widower		Reference	Reference
Education			
Primary and lower		6.05 (4.65 to 7.88)	5.64 (4.32 to 7.37)
Secondary		2.06 (1.57 to 2.70)	1.96 (1.50 to 2.58)
College and above		Reference	Reference
Wealth quintile			
Poorest		1.50 (1.26 to 1.78)	1.48 (1.25 to 1.77)
Poor		1.48 (1.24 to 1.77)	1.47 (1.23 to 1.75)
Average		1.16 (0.97 to 1.38)	1.16 (0.97 to 1.38)
Rich		1.14 (0.96 to 1.35)	1.14 (0.96 to 1.36)
Richest		Reference	Reference
Social capital		0.95 (0.92 to 0.98)	0.95 (0.92 to 0.98)
Smoking			
Smoker			1.12 (0.95 to 1.31)
Past smoker			1.07 (0.86 to 1.34)
Non-smoker			Reference
Chronic diseases			0.95 (0.87 to 1.03)
Depression			1.03 (1.02 to 1.04)

SES, socioeconomic status.

To investigate the relationship between childhood health and childhood SES, we conducted a χ² test and computed a tetrachoric correlation (see online supplemental table S6). A higher proportion of individuals classified in the ‘poor SES’ group reported ‘unhealthy’ childhood (9.43%) compared with those in the ‘non-poor SES’ group (6.69%). The χ² test confirmed a statistically significant association between them (χ²=6.09, p=0.014). In addition, the tetrachoric correlation (ρ=0.094, SE=0.037, p=0.013) indicated a modest but significant positive relationship, suggesting that ‘poor SES’ childhood was associated with an increased likelihood of experiencing ‘unhealthy’ childhood health. We further assessed the interaction between childhood health and SES on dementia risk in table 4. In logistic regression models fully adjusted for age, sex, employment, marital status, education, wealth, social capital, smoking, chronic diseases and depression, poor SES was associated with increased dementia risk without interaction (p=0.048), but this effect was not significant with interaction (p=0.052). Unhealthy childhood showed non-significant effects both without interaction (p=0.061) and with interaction (p=0.496). The multiplicative interaction was not significant (OR=0.88, 95% CI: 0.30 to 2.57, p=0.809), nor were additive interactions, measured by relative excess risk due to interaction (RERI=−0.06, p=0.935), attributable proportion (AP=−0.04, p=0.935) and synergy index (S=0.93, p=0.259). These results suggest that the interaction between unhealthy childhood and poor SES does not significantly amplify the dementia risk beyond their individual effects.

**Table 4:** Multiplicative and additive interactions between childhood health and childhood SES on dementia risk

Interaction type	Measure	Estimate (95% CI)	P value
Multiplicative interaction (OR scale)			
	Unhealthy childhood (without interaction)	1.27 (0.99 to 1.63)	0.061
	Poor SES (without interaction)	1.42 (1.00 to 2.01)	0.048
	Unhealthy childhood (with interaction)	1.44 (0.51 to 4.09)	0.496
	Poor SES (with interaction)	1.44 (1.00 to 2.09)	0.052
	Interaction term	0.88 (0.30 to 2.57)	0.809
Additive interaction (coefficient scale)			
	RERI	−0.06 (−1.59 to 1.47)	0.935
	AP	−0.04 (−0.88 to 0.81)	0.935
	S	0.93 (−0.68 to 2.54)	0.259

AP, attributable proportion; RERI, relative excess risk due to interaction; S, synergy index; SES, socioeconomic status.

The sensitivity analysis using imputed data yielded results consistent with the main findings, reaffirming the associations between childhood health, childhood SES and dementia risk in later life (see online supplemental tables S7 and S8). These findings reinforce the robustness of the original results and suggest that missing data did not substantially affect the observed associations.

## Discussion

This study investigated the association between childhood health, childhood SES and the risk of dementia in later life among older adults in Indonesia, using data from the nationally representative Indonesia Family Life Survey. Guided by a life course perspective, our analysis aimed to determine whether early-life disadvantage, specifically poor health and low SES, shapes dementia risk in old age.

Our findings showed that 15.67% of the sample were at high risk of dementia, which is notably lower than the 27.9% prevalence reported in another cross-sectional study by Farina *et al* conducted in Indonesia.^32^ This discrepancy may be explained by differences in the age groups studied. While their study focused exclusively on individuals aged 65 and above, our study included participants aged 50 and over. Given that dementia risk increases with age,^33^ the older sample in their study likely contributed to the higher prevalence observed.

Nonetheless, the prevalence in our sample remains relatively high compared with similar studies in high-income countries. For instance, a study using the English Longitudinal Study of Ageing (ELSA) reported a dementia prevalence of 9.7% among adults aged 70 and over,^34^ while the US HRS found a prevalence of 11.2% in a similar age group.^34^ A study in Sweden estimated a dementia prevalence of 12.5% among adults aged 65 and older.^35^ The higher prevalence in our Indonesian sample compared with these high-income countries likely reflects the cumulative impact of socioeconomic and health-related disadvantages prevalent in LMICs, such as Indonesia.^32^ Limited access to formal education, higher rates of childhood malnutrition, inadequate preventive healthcare and increased exposure to infectious and non-communicable diseases may contribute to an earlier onset and elevated risk of dementia.^36^ These factors, compounded across the life course, create a higher burden of dementia risk in Indonesia compared with high-income settings with better healthcare infrastructure and socioeconomic conditions.

### Childhood health and dementia risk

This study found a statistically significant association between childhood health and dementia risk in later life. Participants with ‘unhealthy’ childhood health had 1.17 times higher odds of being at high risk of dementia.

Nonetheless, relatively few studies have explicitly examined the relationship between childhood health and dementia risk. Among them, Kobayashi *et al* adopted a similar conceptualisation of childhood health, using a retrospective self-rated measure, and found that individuals who reported poor childhood health were significantly more likely to experience dementia risk in later life.^27^ Other studies have investigated components of childhood health that likely contribute to dementia risk. In China, Zhang and colleagues found that short arm span, lower knee height and poor childhood nutrition were linked to the risk of dementia in later life.^28^ Similarly, a US study by Case and Paxson demonstrated that children who experienced a higher early-life disease burden were significantly more likely to have lower cognitive test performance in later adulthood.^37^

The mechanisms linking unhealthy childhood health to increased dementia risk are multifaceted. Childhood is a critical period for brain development, and inadequate nutrition or frequent illnesses during this time can cause lasting damage to brain structure and function, leading to long-term cognitive deficits and an increased risk of dementia.^38^ Chronic childhood conditions may also trigger persistent inflammation and oxidative stress, processes that contribute to neurodegeneration.^39^ Furthermore, these health challenges often lead to social difficulties, such as isolation and stigma, which reduce social engagement, a known protective factor against dementia.^40^ Research indicates that the impact of childhood chronic diseases may accelerate the progression of brain pathology, causing dementia symptoms to emerge sooner.^41^,^43^ These biological and social factors provide a plausible explanation for the lasting effect of poor childhood health on the increased risk of dementia.

A key challenge in this area of research, however, lies in the lack of a standardised definition for childhood health. In our study, childhood health was measured using a multidimensional approach incorporating both subjective and objective indicators (see Methods). This strategy aimed to reflect the cumulative nature of childhood health disadvantage. In contrast, other studies have relied on a single self-reported item or narrow proxies.^27 28^ Without a shared framework for defining and measuring childhood health, comparisons across studies remain difficult, and the accumulation of coherent evidence is limited.

### Childhood SES and dementia risk

In addition to childhood health, childhood SES was also significantly associated with dementia risk. Individuals from poor childhood SES backgrounds had 1.39 times higher odds of dementia risk in later life after adjusting for covariates. These findings strengthen the growing body of evidence linking childhood poverty to poorer cognitive outcomes in later life. Studies conducted across diverse contexts, including China,^44^ Finland,^9^ Sweden^45^ and the USA,^46^ have consistently shown that low childhood SES is associated with lower cognitive ability in older adulthood.

However, the relationship between childhood SES and dementia risk is complex. A longitudinal study in Sweden found that the effect of childhood SES on later cognitive ability was largely explained by genetic factors.^47^ Research from the UK suggests that childhood socioeconomic conditions influence cognitive ability primarily through indirect pathways, especially via educational attainment; when education is accounted for, no direct association between childhood SES and mid-life cognition remains.^48^ These findings were reinforced by a further UK study that reported no direct effect of childhood socioeconomic conditions on adult cognitive function.^49^ These findings suggest that the influence of childhood SES on dementia risk may operate through both direct and indirect mechanisms, highlighting the need for further research to disentangle these mechanisms.

As with childhood health, a key methodological challenge in this literature is the lack of standardised definitions of childhood SES. For instance, Zhang *et al* measured childhood SES using parental education and father’s occupation;^44^ whereas, Luo and Waite additionally included subjective family financial well-being alongside parental education (≥8 years) and father’s white-collar occupation.^46^ To facilitate more consistent and comparable research in the future, it is essential to establish and use a unified, standardised and multidimensional definition of childhood SES in studies examining dementia risk.

### Later-life covariates

As discussed above, the relationship between childhood SES and dementia risk may be partly mediated by later-life factors. Although this study focused on childhood health and childhood SES, several adulthood characteristics were also significantly associated with dementia risk and offer important contextual insights.

Older age remained a strong predictor of dementia risk, consistent with biological pathways such as neurodegeneration and the accumulation of beta-amyloid and tau proteins.^50^ Education emerged as a key protective factor, potentially serving both as a form of cognitive reserve and as an indirect pathway through which childhood SES lowers later dementia risk.^51^ Participants in lower wealth quintiles faced elevated dementia risk, suggesting that socioeconomic disadvantage across the life course may compound early-life vulnerabilities.^52^ Furthermore, the inclusion of depression in the model attenuated the association between childhood health and dementia risk to non-significance. This finding suggests that later-life depression may partially mediate the long-term impact of childhood health on dementia risk, consistent with findings from other studies.^53 54^

### Interaction between childhood health and SES

The modest but statistically significant association between poor childhood SES and unhealthy childhood health supports the hypothesis that early-life socioeconomic disadvantage contributes to adverse health conditions.^36^ This likely reflects limited access to adequate nutrition, sanitation and healthcare during early life, particularly among cohorts born in the mid-20th century when poverty and malnutrition were widespread across Indonesia.^32^ While both poor childhood SES and unhealthy childhood health were individually associated with increased dementia risk, our findings did not reveal significant multiplicative or additive interactions between them. This suggests that their effects on dementia risk are independent rather than synergistic. The absence of significant interaction may reflect the multifactorial nature of dementia development and the influence of mediating factors across the life course, such as adult SES, health behaviours and access to care.^1^

To our knowledge, no previous study has explicitly examined the interaction between childhood health and childhood SES with dementia risk. Most prior research has either used composite early-life adversity indices or assessed childhood health and childhood SES as independent predictors.^911 55^,^57^ Although our study did not find a statistically significant interaction, future research should model childhood health and childhood SES as separate but interacting domains, as this clarifies whether dementia risk in later life is driven primarily by childhood health, childhood SES or their combined effect.

### Strengths and limitations

This study offers several strengths. First, it draws on data from the IFLS, a large, nationally representative dataset, which enhances the generalisability of the findings to the broader Indonesian population and other LMICs. Second, the study is among the first to analytically separate childhood health and childhood SES, while also examining their interaction with dementia risk. This distinction contributes novel insights to the life course literature, which has often treated early-life adversity as a single composite measure. Finally, the use of LCA allowed us to construct data-driven clusters of childhood health and SES, helping to reduce misclassification and potential recall bias associated with self-reported retrospective data.

However, several limitations should be acknowledged. First, dementia risk was inferred from a brief cognitive test (TICS) rather than clinical diagnosis. While this approach is commonly used in large-scale population studies, the TICS-based cut-off points have not been formally validated in the Indonesian context. We recommend that future studies involve clinical experts and undertake empirical validation to establish context-specific criteria. Incorporating clinical diagnostic confirmation would further enhance the reliability and validity of future research. Second, the cross-sectional design limits establishing causality. Longitudinal data tracking cognitive trajectories from childhood onward would be ideal for clarifying causality. Third, survival bias may be present, as individuals who experienced severe early-life adversity and died before older age are excluded. Lastly, the study relies on retrospective self-reports of childhood health and SES information, which may be subject to recall bias. Although LCA mitigates this to some extent, prospective or historical records would provide greater accuracy.

## Conclusion

This study provides robust evidence that both childhood health and childhood SES are independently associated with dementia risk in later life. However, we found no significant interaction between these two early-life factors, suggesting that their effects on dementia risk operate independently rather than synergistically. These findings support a life-course approach to cognitive ageing and underscore the need to address early-life adversity as part of dementia prevention efforts. Standardising definitions of childhood health and childhood SES will be crucial for enhancing comparability across studies. Drawing on nationally representative data from Indonesia, this analysis offers valuable insights for future research and policy development in similar LMICs. In addition, the development of locally validated cognitive assessment tools is essential to improve diagnostic accuracy and ensure culturally appropriate measurement of dementia risk in diverse settings.

## Supplementary material

10.1136/bmjopen-2024-093896 online supplemental file 1

## Data Availability

Data are available in a public, open access repository.
