# Supplementary material for: Association of childhood health and socioeconomic status with dementia risk in older age: a cross-sectional study using the Indonesia Family Life Survey 2014–2015
Source: BMJ Open. 2025 Aug 16;15(8):e093896. doi: 10.1136/bmjopen-2024-093896 (PMC12359521; doi:10.1136/bmjopen-2024-093896)
Supplement: online supplemental file 1 [file bmjopen-15-8-s001.docx]

**Supplemental Material**

Figure S1. Statistical analysis

**Step 1**

**Step 2**

**Step 3**

| **Aim** |  | Identify childhood health and SES clusters |  | Identify the trait of clusters and covariates |  | Identify the relation between childhood health, childhood SES and probable dementia |
| --- | --- | --- | --- | --- | --- | --- |
|  |  |  |  |  |  |  |
| **Method** |  | Latent Class Analysis |  | Descriptive analysis, bivariate analysis |  | Regression analysis in three models:  - Model 1: adjusted for age and sex - Model 2: added employment, marital status, education level, wealth quintile, and social activities - Model 3: added smoking status, number of chronic diseases, and depression scores |
|  |  |  |  |  |  |  |
| **Output** |  | Two clusters of childhood health: 'healthy' and 'unhealthy'; Two clusters of childhood SES: 'poor' and 'non-poor' |  | Frequency and percentage for categorical variables, means and standard deviations for continuous variables |  | Coefficient and 95% CI |

Table S2. Descriptive characteristics of the analytic sample (N = 6,693) and missing data

| Variables | Analytic sample  N=6693 | Missing data (%) |
| --- | --- | --- |
| TICS score, mean (SD) | 12.79 ± 4.19 | 884 (13.21) |
| Dementia (%) |  | 884 (13.21) |
| Normal | 2,747 (47.29) |  |
| CIND | 2,152 (37.05) |  |
| High risk of dementia | 910 (15.67) |  |
| Age, mean (SD) | 59.88 ± 8.10 | 0 |
| Sex, frequency (%) |  | 0 |
| Female | 3,143 (46.96) |  |
| Male | 3,550 (53.04) |  |
| Marital status, frequency (%) |  | 0 |
| Single | 70 (1.05) |  |
| Married | 5,008 (74.82) |  |
| Separated | 250 (3.74) |  |
| Widower | 1,365 (20.39) |  |
| Education, frequency (%) |  | 12 (0.18) |
| Primary and lower | 4,583 (68.47) |  |
| Secondary | 1,572 (23.49) |  |
| College and higher | 526 (7.86) |  |
| Employment, frequency (%) |  | 0 |
| No | 2,137 (31.93) |  |
| Yes | 4,556 (68.07) |  |
| Wealth quintile, frequency (%) |  | 491 (7.34) |
| Poorest | 1,172 (17.51) |  |
| Poor | 1,240 (18.53) |  |
| Average | 1,272 (19.00) |  |
| Rich | 1,247 (18.63) |  |
| Richest | 1,271 (18.99) |  |
| Social capital, mean (SD) | 2.22 ± 1.96 | 0 |
| Smoking, frequency (%) |  | 0 |
| Smoker | 2,167 (32.38) |  |
| Past smoker | 634 (9.47) |  |
| Non-smoker | 3,892 (58.15) |  |
| Chronic diseases, mean (SD) | 0.41 ± 0.66 | 0 |
| Depression, mean (SD) | 15.65 ± 4.69 | 0 |

Table S3. Latent Class Analysis results

| Latent class models | Class  N (percent) | | | AIC | BIC | Entropy |
| --- | --- | --- | --- | --- | --- | --- |
|  | **1** | **2** | **3** |  |  |  |
| Childhood health | | | | | | |
| Model 1: Two classes | 610 (9.11) | 6,083 (90.89) |  | 26453.71 | 26528.61 | 0.95 |
| Model 2: Three classes | 689 (10.29) | 5,982 (89.38) | 22 (0.33) | 26447.80 | 26563.55 | 0.90 |
| Childhood SES | | | | | | |
| Model 1: Two classes | 5,931 (88.61) | 762 (11.39) |  | 26963.54 | 27038.44 | 0.96 |
| Model 2: Three classes | 3,172 (47.39) | 2,759 (41.22) | 762 (11.39) | 26944.03 | 27059.78 | 0.84 |

Table S4. Characteristics of observed variables between two latent classes of childhood health

|  | **Healthy** | **Unhealthy** |
| --- | --- | --- |
|  | **N = 6,083 (%)** | **N = 610 (%)** |
| Childhood health |  |  |
| Good and better | 4,052 (66.61) | 258 ( 42.30) |
| Fair and poor | 2,031 (33.39) | 352 (57.70) |
| Were ever absent from school ≥ 1 month | 1,018 (16.74) | 485 (79.51) |
| Were ever confined to bed ≥ 1 month | 79 (1.30) | 591 (96.89) |
| Were ever hospitalised ≥ 1 month | 37 (0.61) | 96 (15.74) |
| Were ever hunger | 929 (15.27) | 198 (32.46) |

Table S5. Characteristics of observed variables between two latent classes of childhood SES

|  | **Non-poor SES** | **Poor SES** |
| --- | --- | --- |
|  | **N = 762 (%)** | **N = 5,931 (%)** |
| Had overcrowding | 614 (80.90) | 5,002 (85.11) |
| Had electricity | 739 (96.98) | 172 (2.90) |
| Had running water | 409 (53.67) | 292 (4.92) |
| Had own toilet | 664 (87.14) | 1,644 (27.72) |
| Had more than ten books | 265 (34.78) | 445 (7.50) |

Table S6. Association between childhood health and childhood SES

| Childhood health | Non-poor childhood SES | Poor childhood SES | Total |
| --- | --- | --- | --- |
| Healthy | 711 (93.31%) | 5,372 (90.57%) | 6,083 (90.89%) |
| Unhealthy | 51 (6.69%) | 559 (9.43%) | 610 (9.11%) |
| Total | 762 (11.39%) | 5,931 (88.61%) | 6,693 (100%) |
| Chi-squared test: χ²(1) = 6.09, *p* = 0.014 | |  |  |
| Tetrachoric correlation: ρ = 0.094, SE = 0.037, *p* = 0.013 | | |  |

Table S7. Multivariable logistic regression results showing the association between childhood health and dementia risk outcome, adjusted for covariates with imputed missing data

|  | **Model 1**  **OR (95% CI)** | **Model 2**  **OR (95% CI)** | **Model 3**  **OR (95% CI)** |
| --- | --- | --- | --- |
| Clusters |  |  |  |
| Healthy | reference | reference | reference |
| Unhealthy | 1.25 (1.06; 1.48) | 1.17 (0.99; 1.39) | 1.13 (0.95; 1.34) |
| Age | 1.06 (1.05; 1.06) | 1.05 (1.04; 1.06) | 1.05 (1.04; 1.06) |
| Sex |  |  |  |
| Female | 0.81 (0.73; 0.89) | 1.03 (0.92; 1.16) | 0.94 (0.80; 1.11) |
| Male | reference | Reference | Reference |
| Employed |  |  |  |
| Yes |  | Reference | Reference |
| No |  | 0.99 (0.88; 1.12) | 1.00 (0.89; 1.13) |
| Marital status |  |  |  |
| Single |  | 1.21 (0.70; 2.09) | 1.20 (0.70; 2.08) |
| Married |  | 0.98 (0.85; 1.13) | 0.99 (0.86; 1.14) |
| Separated |  | 1.08 (0.80; 1.45) | 1.06 (0.79; 1.43) |
| Widower |  | Reference | Reference |
| Education |  |  |  |
| Primary and lower |  | 6.38 (5.01; 8.12) | 5.95 (4.67; 7.58) |
| Secondary |  | 2.04 (1.59; 2.61) | 1.95 (1.52; 2.50) |
| College and higher |  | reference | reference |
| Wealth quintile |  |  |  |
| Poorest |  | 1.52 (1.27; 1.81) | 1.50 (1.26; 1.79) |
| Poor |  | 1.54 (1.31; 1.82) | 1.53 (1.30; 1.81) |
| Avarage |  | 1.18 (1.00; 1.39) | 1.17 (0.99; 1.38) |
| Rich |  | 1.16 (0.98; 1.38) | 1.17 (0.99; 1.38) |
| Richest |  | Reference | reference |
| Social capital |  | 0.95 (0.92; 0.98) | 0.95 (0.92; 0.98) |
| Smoking |  |  |  |
| Smoker |  |  | 1.14 (0.97; 1.34) |
| Past smoker |  |  | 1.08 (0.88; 1.34) |
| Non-smoker |  |  | reference |
| Chronic diseases |  |  | 0.95 (0.87; 1.03) |
| Depression |  |  | 1.03 (1.02; 1.04) |

Table S8. Multivariable logistic regression results showing the association between childhood SES and dementia risk outcome, adjusted for covariates with imputed missing data

|  | **Model 1**  **OR (95% CI)** | **Model 2**  **OR (95% CI)** | **Model 3**  **OR (95% CI)** |
| --- | --- | --- | --- |
| Clusters |  |  |  |
| Non-poor SES | reference | reference | reference |
| Poor SES | 2.58 (2.18; 3.05) | 1.37 (1.14; 1.64) | 1.37 (1.14; 1.64) |
| Age (years) | 1.05 (1.05; 1.06) | 1.05 (1.04; 1.05) | 1.05 (1.04; 1.06) |
| Sex |  |  |  |
| Female | 0.80 (0.72; 0.88) | 1.02 (0.91; 1.15) | 0.93 (0.79; 1.10) |
| Male | reference | Reference | Reference |
| Employed |  |  |  |
| Yes |  | Reference | Reference |
| No |  | 1.00 (0.89; 1.13) | 1.01 (0.89; 1.14) |
| Marital status |  |  |  |
| Single |  | 1.25 (0.72; 2.16) | 1.24 (0.72; 2.15) |
| Married |  | 0.98 (0.85; 1.12) | 0.99 (0.86; 1.14) |
| Separated |  | 1.08 (0.81; 1.46) | 1.06 (0.79; 1.43) |
| Widower |  | reference | reference |
| Education |  |  |  |
| Primary and lower |  | 5.95 (4.66; 7.59) | 5.53 (4.33; 7.08) |
| Secondary |  | 1.99 (1.56; 2.55) | 1.90 (1.49; 2.44) |
| College and above |  | Reference | Reference |
| Wealth quintile |  |  |  |
| Poorest |  | 1.48 (1.24; 1.77) | 1.47 (1.23; 1.76) |
| Poor |  | 1.51 (1.28; 1.78) | 1.50 (1.28; 1.77) |
| Avarage |  | 1.16 (0.98; 1.37) | 1.15 (0.97; 1.36) |
| Rich |  | 1.14 (0.97; 1.35) | 1.15 (0.97; 1.36) |
| Richest |  | Reference | reference |
| Social capital |  | 0.95 (0.92; 0.98) | 0.95 (0.92; 0.97) |
| Smoking |  |  |  |
| Smoker |  |  | 1.15 (0.98; 1.35) |
| Past smoker |  |  | 1.09 (0.88; 1.34) |
| Non-smoker |  |  | reference |
| Chronic diseases |  |  | 0.95 (0.87; 1.03) |
| Depression |  |  | 1.03 (1.02; 1.04) |
