# Supplementary material for: Association of childhood health and socioeconomic status with dementia risk in older age: a cross-sectional study using the Indonesia Family Life Survey 2014–2015
Source: BMJ Open. 2025 Aug 16;15(8):e093896. doi: 10.1136/bmjopen-2024-093896 (PMC12359521; doi:10.1136/bmjopen-2024-093896)
Supplement: Supplementary file 2 [file bmjopen-2024-093896.reviewer_comments.pdf]

PEER REVIEW HISTORY

BMJ Open publishes all reviews undertaken for accepted manuscripts. Reviewers are asked to complete a checklist review form and are provided with free text boxes to elaborate on their assessment. These free text comments are reproduced below.

ARTICLE DETAILS

Title (Provisional)

Association of Childhood Health and Socioeconomic Status with Dementia Risk in Older Age: A Cross-Sectional Study Using the Indonesia Family Life Survey 2014–2015

Authors

Le, Tung; Lee, Amanda; Gilleen, James; Maharani, Asri

VERSION 1 - REVIEW

**Reviewer**	**1**
**Name**	**Bodryzlova, Yuliya**
**Affiliation**	**Institut National de Psychiatrie Légale Philippe-Pinel, Montreal, Canada**
**Date**	**21-Jan-2025**
**COI**	**None**

Dear authors, editors,

Thank you for the opportunity to revise this work devoted to the understanding of the association between childhood health state and socioeconomic conditions and cognitive health outcomes.
Unfortunately, besides some interesting collateral findings, the work has important methodological caveats which, in my opinion, make it unpublishable in its current state.

Below is the list of comments on the

manuscript. Best regards.

Please find a proper term for your outcome. Across the text, you use several different labels that have very different meanings.

Please format the reference list according to existing rules.

Abstract.

Findings: Respondents in the 'unhealthy' cluster had a 1.95 times higher risk of CIND and probable dementia than those in the 'healthy' cluster (95% CI: 1.10, 3.45). The 'poor SES' cluster had a 1.47 times higher risk than the 'non-poor' cluster (95% CI: 1.20, 2 1.80). Age,

education, wealth, social capital, and depression were also linked to higher risk. Please report findings according to the fully adjusted models in Tables 2 and 3.

Introduction.

Dementia is a crucial and escalating global health issue, affecting over 50 million people 3 worldwide, with around 10 million new cases annually (1). Please use references in peer-reviewed literature.

By 2050, the number of people 4 with dementia is projected to exceed 150 million (2). This condition includes cognitive impairments such as memory loss, difficulties in thinking and problem-solving, and 6 behavioural changes that disrupt daily life (3). Please verify the definitions and correct them accordingly.

Factors such as malnutrition, exposure to stress, and limited access to education and healthcare contributed to this negative association (8). Nutritional deficiencies and restricted healthcare access further exacerbate these challenges (9). Please avoid repetitions.

Data from the 25 10/66 Dementia Research Group, covering Latin America, Africa, and Asia, also link 26 childhood poverty and malnutrition to accelerated cognitive decline in old age (10). Please provide a reference to more specific findings

However, there is a gap in research examining both childhood health and socioeconomic status (SES) together in relation to dementia, which this study aims to address. Identifying a significant link would support the development of targeted interventions and policies to reduce probable dementia from a life- course perspective. Please revise the existing literature on the topic and rewrite your entire introduction in light of this revision.

Methods

Present key elements of study design early in the paper Describe the setting, locations, and relevant dates, including periods of recruitment, exposure, follow-up, and data collection

Give the eligibility criteria, and the sources and methods of selection of participants. Describe methods of follow-up.

For each variable of interest, give sources of data and details of methods of assessment (measurement). Describe comparability of assessment methods if there is more than one group

We classified the participants into three groups based on their total scores: 12-27 20 points (normal cognitive function), 7-11 points (at risk for dementia or cognitive 21 impairment), and 0-6 points (high risk for dementia). Please provide the sensitivity and specificity of this method of dementia detection.

Explain how missing data were addressed

Finally, regression analyses estimated the associations between childhood health- please describe the type of regression

Explain how loss to follow-up was

addressed Results

Your participation rate is about 20%? (6 000 out of 30 000 participants?) Please report the

numbers of individuals at each stage of the study—eg numbers potentially eligible, examined for eligibility, confirmed eligible, included in the study, completing follow-up, and analysed. Give reasons for non- participation at each stage. Consider use of a flow diagram. Give characteristics of study participants (eg demographic, clinical, social) and information on exposures and potential confounders. Indicate number of participants with missing data for each variable of interest. Cohort study—Summarise follow-up time (eg, average and total amount)

Table 1 highlights gender, education, SES, and health disparities in Indonesia. In Indonesia or in your sample?

We classified the participants into three groups based on their total scores: 12-27 points (normal cognitive function), 7-11 points (at risk for dementia or cognitive 21 impairment), and 0-6 points (high risk for dementia). You have three categories of outcomes, what kind of regression did you construct? Why are you reporting

To isolate net associations with probable dementia, we built three regression models, and 22 the findings are shown below 1) The term "Probable dementia" refers to a specific health condition, which is not a part of your reported results. Please correct accordingly. 2) Please indicate that Table 2 reports the results on the association with the child-age health status and Table 3 with the child-age socioeconomic status.

The most crucial change concerned the childhood health clusters. In Table 2, the association between an ‘unhealthy’ childhood and probable dementia was not significant and this lack of significance continued when the childhood health-SES interaction was excluded. Unclear. If you would like to report the interaction between childhood poverty and ill health, please report your findings according to the recent guidelines on interaction studies.

Discussion

The findings revealed a significant link, with individuals who had poor childhood health being nearly twice as likely to develop dementia compared to those with healthier childhoods. Please correct according to the results presented in Table 3.

Overall, the discussion is unspecific to findings and the study questions, and, alike the introduction, represents a set of pieces of general knowledge of neurodegenerative diseases.

**Reviewer**	**2**
**Name**	**Osaki, Tohmi**
**Affiliation**	**Kobe Gakuin University Faculty of Rehabilitation, Department of Occupational Therapy**
**Date**	**24-Apr-2025**
**COI**	**None**

This study analyzed data collected from 6,693 Indonesians between 2014 and 2015 to examine whether health and socioeconomic conditions in childhood are associated with

dementia in later life. The authors suggest the importance of a good education from an early age, which is a helpful suggestion that applies to many other countries as well.

However, the manuscript lacked some important information, making a thorough review difficult. In this review, I will refrain from mentioning minor points and instead ask the authors for their opinions on the major points raised.

Primary outcome

The primary outcome of this study is the TICS score, but there is no description of the score distribution or interpretation. What percentage of participants were classified as CIND or probable dementia, and was this appropriate compared to other data from Indonesia? Furthermore, although the target population was 60 or younger (average), considering the incidence rate of dementia in that age group in Indonesia, was this classification appropriate? In the TICS-27 adopted in this study, memory and calculation tasks account for a large proportion of the score distribution. These two tests are strongly influenced by educational background, even in people without dementia. Why didn't the authors adopt other versions of TICS? Although low cognitive function can undoubtedly be a risk factor for dementia due to low cognitive reserve, it is not referred to as "cognitive decline" or "dementia" unless there is a decline.

Also, I feel uncomfortable with the manuscript's conclusion that "the unhealthy group had a 1.95 times higher risk of CIND and probable dementia." This interpretation is statistically incorrect. This study shows OR, not RR, so simply expressing the risk as XX times higher is inappropriate.

Objectives

What do the authors want to emphasize in this study? From the Abstract to the Methods section, it is the relationship between health and socioeconomic status in childhood and cognitive function in later life. However, the Discussion section is mainly devoted to discussing other secondary outcomes. The discussion seemed to be geared toward the pre-determined conclusion that "improving education is important." Statistically, there is also the problem of multiple testing. It is recommended that the authors revisit their research design, including statistical analysis, focusing on what they wish to demonstrate.

VERSION 1 - AUTHOR RESPONSE

REVIEWER 1 COMMENTS

<u>Comments from Reviewer #1</u>

Please find a proper term for your outcome. Across the text, you use several different labels that have very different meanings.

Authors' response

Thank you for your helpful feedback. To ensure clarity and consistency, we have now standardised the term across the manuscript. We exclusively use the term "dementia risk" (or "risk of dementia") to refer to the study outcome.

Comments from Reviewer #1

Please format the reference list according to existing rules.

Authors' response

Thank you for your comment. We have reformatted the reference list according to the Vancouver style, as specified in the BMJ Open Instructions for Authors. We used EndNote referencing software to ensure consistency and adherence to the journal's citation guidelines.

Comments from Reviewer #1

Abstract.

Findings: Respondents in the 'unhealthy' cluster had a 1.95 times higher risk of CIND and probable dementia than those in the 'healthy' cluster (95% CI: 1.10, 3.45). The 'poor SES' cluster had a 1.47 times higher risk than the 'non-poor' cluster (95% CI: 1.20, 2 1.80). Age, education, wealth, social capital, and depression were also linked to higher risk.

Please report findings according to the fully adjusted models in Tables 2 and 3.

Authors' response

Thank you for your comment. We have revised the Results section of the Abstract to report findings according to the fully adjusted models in Tables 2 and 3, as requested (Abstract, Results, page 3, lines 9-13) :

"***Results*** *Individuals in the 'unhealthy' childhood health cluster had 1.17 times higher odds of dementia risk compared to the 'healthy' cluster (95% CI: 1.00–1.38), a borderline*

association, while those in the 'poor socioeconomic status' cluster had 1.39 times higher odds compared to the 'non-poor' cluster (95% CI: 1.15–1.68)."

Comments from Reviewer #1

Introduction.

Dementia is a crucial and escalating global health issue, affecting over 50 million people 3 worldwide, with around 10 million new cases annually (1).

Please use references in peer-reviewed literature.

Authors' response

Thank you for your feedback. We have removed the data from WHO and added the reference in peer-reviewed literature (Introduction, page 5, lines 1-3):

"Introduction

Dementia is a major global public health concern because it progressively worsens over time and currently has no cure 1"

1. Livingston G, Huntley J, Sommerlad A, et al. Dementia prevention, intervention, and care: 2020 report of the Lancet Commission. Lancet 2020;396(10248):413-46. doi: 10.1016/s0140-6736(20)30367-6

Comments from Reviewer #1

By 2050, the number of people 4 with dementia is projected to exceed 150 million (2). This condition includes cognitive impairments such as memory loss, difficulties in thinking and problem-solving, and 6 behavioural changes that disrupt daily life (3).

Please verify the definitions and correct them accordingly.

Authors' response

Thank you for your thoughtful feedback. We have verified the definition used in peer-reviewed literature and corrected it accordingly (Introduction, page 5, lines 3-4):

"It is a chronic and progressive syndrome characterised by deterioration in memory, thinking, behaviour, and the ability to perform everyday activities 2."

2. Cipriani G, Danti S, Picchi L, et al. Daily functioning and dementia. Dement Neuropsychol 2020;14(2):93-102. doi: 10.1590/1980-57642020dn14-020001

Comments from Reviewer #1

Factors such as malnutrition, exposure to stress, and limited access to education and healthcare contributed to this negative association (8). Nutritional deficiencies and restricted healthcare access further exacerbate these challenges (9).

Please avoid repetitions.

Authors' response

Thank you for your helpful feedback. We have removed the repetition.

Comments from Reviewer #1

Data from the 25 10/66 Dementia Research Group, covering Latin America, Africa, and Asia, also link 26 childhood poverty and malnutrition to accelerated cognitive decline in old age (10).

Please provide a reference to more specific findings.

Authors' response

Thank you for your suggestion. We have added some references to more specific findings. For example, on page 5, lines 12-14, we now include:

"In Malaysia, Momtaz et al. found that childhood food insufficiency, measured through recurrent experiences of hunger, predicted an 81% higher risk of dementia in older adulthood 7."

Comments from Reviewer #1

However, there is a gap in research examining both childhood health and socioeconomic status (SES) together in relation to dementia, which this study aims to address. Identifying a

significant link would support the development of targeted interventions and policies to reduce probable dementia from a life-course perspective.

Please revise the existing literature on the topic and rewrite your entire introduction in light of this revision.

Authors' response

Thank you for your valuable feedback. We have rewritten the Introduction section entirely to address the points raised. The revised Introduction now:

- Uses references exclusively from peer-reviewed literature.
- Provides a clear, accurate definition of dementia based on peer-reviewed literature.
- Removes repetitive statements.
- Includes more specific findings and relevant studies.
- Expands the review of existing literature to reflect diverse and sometimes conflicting evidence on childhood health, childhood socioeconomic status, and dementia risk.
- Identifies research gaps, especially the lack of studies examining childhood health and SES together.

We hope these revisions address your concerns and improve the clarity and quality of the Introduction.

Comments from Reviewer #1

Methods

Present key elements of study design early in the paper.

Authors' response

Thank you for your helpful comment. We have addressed this by clearly including the study design in both the title and the abstract, ensuring it is presented early and prominently in the manuscript.

Comments from Reviewer #1

Describe the setting, locations, and relevant dates, including periods of recruitment, exposure, follow-up, and data collection.

Give the eligibility criteria, and the sources and methods of selection of participants. Describe methods of follow-up.

Authors' response

Thank you for your comment. We have clarified in the Methods section that our study uses anonymous, publicly available data from Wave 5 of the Indonesia Family Life Survey (IFLS). The IFLS covers multiple provinces across Indonesia, and the Wave 5 data were collected between 2014 and 2015. Although the IFLS is a longitudinal survey, our analysis is restricted to this single wave. Therefore, the study design is cross-sectional without follow-up data. We have detailed the setting, locations, and data collection period accordingly (Methods, Study Design and Participants, page 6, lines 18-25).

"Study Design and Participants

This cross-sectional study utilised anonymous, public data from Wave 5 (2014–2015) of the Indonesia Family Life Survey (IFLS), a nationally representative longitudinal survey 14. The IFLS collects comprehensive socioeconomic and health information through face-to-face interviews and is part of the Health and Retirement Study (HRS) family of harmonised ageing surveys conducted across multiple countries 15. Wave 5 was selected for this study because it was the first to include detailed retrospective self-reported information on childhood health and SES, essential for our research objectives."

Comments from Reviewer #1

For each variable of interest, give sources of data and details of methods of assessment (measurement).

Describe comparability of assessment methods if there is more than one group.

Authors' response

Thank you for your comment. We have provided detailed descriptions in the Methods section regarding the sources of data and assessment methods for each variable of interest, including childhood health, childhood SES, dementia, and covariates. As our study includes only one population group, comparability between multiple groups is not applicable.

Comments from Reviewer #1

We classified the participants into three groups based on their total scores: 12-27 20 points (normal cognitive function), 7-11 points (at risk for dementia or cognitive 21 impairment), and 0-6 points (high risk for dementia).

Please provide the sensitivity and specificity of this method of dementia detection.

Authors' response

Thank you for your helpful feedback. We have provided details of the dementia classification method, including the sensitivity and specificity of the risk of dementia detection (Methods, Dementia Measures, page 8, line 25 – page 9, line 6):

"Following the classification approach developed by Langa et al. for the HRS and its international sister studies 22, participants were categorised into three groups based on their total TICS-27 score: 12–27 points indicating normal cognitive function, 7–11 points representing cognitively impaired but not demented (CIND), and 0–6 points signifying a high risk of dementia. These cut-offs have been used in prior studies and shown to have acceptable sensitivity and specificity in distinguishing levels of cognitive function in population-based settings where clinical diagnosis is not available 22 23."

Comments from Reviewer #1

Explain how missing data were addressed.

Authors' response

Thank you for your valuable suggestion. We have added our approach to handling missing data in the revised manuscript (Methods, Sensitivity Analysis, page 10, line 25 – page 11, line 2):

"Sensitivity Analysis

To assess the robustness of the main findings, we conducted a sensitivity analysis using multiple imputation to address missing data, following White et al.'s guidance 27. The regression models were re-estimated using the imputed data and compared with the original results to examine whether the observed associations remained consistent."

Comments from Reviewer #1

Finally, regression analyses estimated the associations between childhood health.

Please describe the type of regression.

Authors' response

Thank you for your comment. We have now specified in the Methods section that multivariate ordinal logistic regression analyses were used (Methods, Statistical Analysis, page 10, lines 12-15).

"Then, multivariate ordinal logistic regression analyses were used to estimate the associations between childhood health, childhood SES, and their interaction with dementia risk within the identified latent classes."

Comments from Reviewer #1

Explain how loss to follow-up was addressed

Authors' response

Thank you for your comment. As our study is cross-sectional and based solely on Wave 5 of the IFLS, there is no follow-up or loss to follow-up to address.

Comments from Reviewer #1

Results

Your participation rate is about 20%? (6 000 out of 30 000 participants?)

Please report the numbers of individuals at each stage of the study—eg numbers potentially eligible, examined for eligibility, confirmed eligible, included in the study, completing follow-up, and analysed. Give reasons for non-participation at each stage. Consider use of a flow diagram.

Authors' response

Thank you for your comment. We have clarified the participant flow with exact numbers in the Methods section (Methods, Study Design and Participants, page 6, line 25 – page 7, line 4):

"From the original Wave 5 sample (N=36,391 participants aged ≥15 years across 13 provinces), we restricted our analysis to adults aged ≥50 years (n=27,909 excluded) with complete childhood health and SES data (n=1,789 excluded), resulting in a final analytical sample of 6,693 participants. This age cutoff ensured capture of the target population at elevated dementia risk while maintaining a sufficient sample size for robust analysis 11 16."

Comments from Reviewer #1

Give characteristics of study participants (eg demographic, clinical, social) and information on exposures and potential confounders. Indicate number of participants with missing data for each variable of interest. Cohort study—Summarise follow-up time (eg, average and total amount).

Authors' response

Thank you for your valuable feedback. As this is a cross-sectional study rather than a cohort study, no follow-up time is applicable. Detailed characteristics of the study participants, including demographics and other relevant variables, are now provided in the supplemental table S2, which also reports the number of participants with missing data for each variable.

Comments from Reviewer #1

Table 1 highlights gender, education, SES, and health disparities in Indonesia.

In Indonesia or in your sample?

Authors' response

Thank you for this helpful comment. We have clarified that Table 1 highlights gender, education, SES, and health disparities in the analytic sample (Results, page 13, line 1):

"Table 1 highlights gender, education, SES, and health disparities in the analytic sample."

Comments from Reviewer #1

We classified the participants into three groups based on their total scores: 12-27 points (normal cognitive function), 7-11 points (at risk for dementia or cognitive 21 impairment), and 0-6 points (high risk for dementia).

You have three categories of outcomes, what kind of regression did you construct? Why are you reporting.

Authors' response

Thank you for your comment. We conducted multivariate ordinal logistic regression to examine the associations between childhood exposures and levels of dementia risk. While it is possible to analyse cognitive scores as a continuous variable, we chose to categorise participants into three clinically meaningful groups based on established cut-offs from previous literature. This categorisation enhances interpretability in public health and clinical contexts and aligns with practical decision-making frameworks. Moreover, it helps address potential non-linear relationships between cognitive scores and dementia risk.

Comments from Reviewer #1

To isolate net associations with probable dementia, we built three regression models, and 22 the findings are shown below

1) The term "Probable dementia" refers to a specific health condition, which is not a part of your reported results. Please correct accordingly.

Authors' response

Thank you for your comment. We have revised the terminology throughout the manuscript to use "high risk of dementia" or "dementia risk" instead of "probable dementia" to more accurately reflect the nature of our outcome measure.

Comments from Reviewer #1

2) Please indicate that Table 2 reports the results on the association with the child-age health status and Table 3 with the child-age socioeconomic status.

Authors' response

Thank you for your comment. We have clarified in the manuscript that Table 2 reports the associations between childhood health status and dementia risk, while Table 3 reports the associations between childhood SES and dementia risk.

Comments from Reviewer #1

The most crucial change concerned the childhood health clusters. In Table 2, the association between an 'unhealthy' childhood and probable dementia was not significant and this lack of significance continued when the childhood health-SES interaction was excluded.
Unclear. If you would like to report the interaction between childhood poverty and ill health, please report your findings according to the recent guidelines on interaction studies.

Authors' response

Thank you for your valuable feedback. We have revised our writing to provide a clearer and more concise explanation following Knol and VanderWeele (2012) (Results, page 17, lines 5-15):

"A notable change, however, was observed in the association involving the 'unhealthy' childhood cluster. As presented in table 2, Model 3, without incorporating the interaction term, the association between an 'unhealthy' childhood and a high risk of dementia was borderline statistically significant, with an odds ratio of 1.17 (95% CI: 1.00, 1.38).

Nonetheless, the inclusion of a childhood health-SES interaction term in the model led to a significant change in this association. With the interaction term included, the odds of being classified as high risk of dementia among individuals with an 'unhealthy' childhood increased from 1.17 to 1.95 compared to those with a 'healthy' childhood (95% CI: 1.10, 3.45) (see online supplemental table S6). This shift indicates that considering the joint influence of childhood health and SES on dementia risk reveals a statistically significant association for 'unhealthy' childhood, which was not apparent when examining these factors separately."

Mirjam J Knol, Tyler J VanderWeele, Recommendations for presenting analyses of effect modification and interaction, International Journal of Epidemiology, Volume 41, Issue 2, April 2012, Pages 514–520, https://doi.org/10.1093/ije/dyr218.

Comments from Reviewer #1

Discussion

The findings revealed a significant link, with individuals who had poor childhood health being nearly twice as likely to develop dementia compared to those with healthier childhoods. Please correct according to the results presented in Table 3.

Overall, the discussion is unspecific to findings and the study questions, and, alike the introduction, represents a set of pieces of general knowledge of neurodegenerative diseases.

Authors' response

Thank you for your helpful comments. We have thoroughly revised and rewritten the entire Discussion section to ensure it is specifically focused on our study findings and directly addresses the study questions.

REVIEWER 2 COMMENTS

<u>Comments from Reviewer #2</u>

Primary outcome

The primary outcome of this study is the TICS score, but there is no description of the score distribution or interpretation. What percentage of participants were classified as CIND or probable dementia, and was this appropriate compared to other data from Indonesia?

<u>Authors' response</u>

Thank you for your insightful comment. We have now included a detailed description of the TICS score distribution and its interpretation in the supplemental table S2, including the percentages of participants classified as normal, CIND, and high risk of dementia. We have also added a comparison with other data from Indonesia in the Discussion (page 18, lines 3-9):

"Our findings showed that 15.67% of the sample were at high risk of dementia, which is notably lower than the 27.9% prevalence reported in another cross-sectional study by Farina et al. conducted in Indonesia 28. This discrepancy may be explained by differences in the age groups studied. While their study focused exclusively on individuals aged 65 and above, our study included participants aged 50 and over. Given that dementia risk increases with age 29, the older sample in their study likely contributed to the higher prevalence observed."

<u>Comments from Reviewer #2</u>

Furthermore, although the target population was 60 or younger (average), considering the incidence rate of dementia in that age group in Indonesia, was this classification appropriate?

<u>Authors' response</u>

Thank you for raising this important point. Yes, the classification was appropriate for our study population aged 50 and above, based on evidence from previous studies with similar

settings, which indicate that cognitive impairment and early dementia symptoms can be detected in this age group. While this classification may seem unusual in countries with high life expectancies, it aligns with the broader literature on dementia risk and cognitive impairment, where many studies include participants aged 50 and over, and some even start at age 45 (Methods, Study Design and Participants, page 6, line 25 – page 7, line 4):

"From the original Wave 5 sample (N=36,391 participants aged ≥15 years across 13 provinces), we restricted our analysis to adults aged ≥50 years (n=27,909 excluded) with complete childhood health and SES data (n=1,789 excluded), resulting in a final analytical sample of 6,693 participants. This age cutoff ensured capture of the target population at elevated dementia risk while maintaining a sufficient sample size for robust analysis (11, 16)."

Comments from Reviewer #2

In the TICS-27 adopted in this study, memory and calculation tasks account for a large proportion of the score distribution. These two tests are strongly influenced by educational background, even in people without dementia. Why didn't the authors adopt other versions of TICS?

Authors' response

Thank you for your comment. We chose the TICS-27 version as it is the only version available in the Indonesia Family Life Survey (IFLS Wave 5), the dataset used in this study. Other TICS variants, such as the TICS-m or education-adjusted scoring protocols, were not included or available in the IFLS dataset. To account for this limitation, we carefully adjusted for educational attainment in all multivariate ordinal logistic regression models.

Comments from Reviewer #2

Although low cognitive function can undoubtedly be a risk factor for dementia due to low cognitive reserve, it is not referred to as "cognitive decline" or "dementia" unless there is a decline.

<u>Authors' response</u>

Thank you for your important comment. We fully acknowledge that low cognitive function alone does not constitute "cognitive decline" or "dementia" unless there is evidence of decline over time. Therefore, in the revised manuscript, we have carefully used the term "dementia risk" rather than asserting a diagnosis of dementia, following the classification approach developed by Langa et al. for the Health and Retirement Study (HRS) and its international sister studies (Methods, Dementia Measures, page 8, line 25 – page 9, line 6):

"Following the classification approach developed by Langa et al. for the HRS and its international sister studies (22), participants were categorised into three groups based on their total TICS-27 score: 12–27 points indicating normal cognitive function, 7–11 points representing cognitively impaired but not demented (CIND), and 0–6 points signifying a high risk of dementia. These cut-offs have been used in prior studies and shown to have acceptable sensitivity and specificity in distinguishing levels of cognitive function in population-based settings where clinical diagnosis is not available (22, 23)."

<u>Comments from Reviewer #2</u>

Also, I feel uncomfortable with the manuscript's conclusion that "the unhealthy group had a 1.95 times higher risk of CIND and probable dementia." This interpretation is statistically incorrect. This study shows OR, not RR, so simply expressing the risk as XX times higher is inappropriate.

<u>Authors' response</u>

Thank you for your valuable feedback. We have amended the wording throughout the manuscript to use "higher odds of" instead of "higher risk of" to accurately reflect the interpretation of the results. For example, in the Abstract (Results, page 3, lines 9-14):

*"**Results** Individuals in the 'unhealthy' childhood health cluster had 1.17 times higher odds of dementia risk compared to the 'healthy' cluster (95% CI: 1.00–1.38), a borderline association, while those in the 'poor socioeconomic status' cluster had 1.39 times higher odds compared to the 'non-poor' cluster (95% CI: 1.15–1.68). When the interaction between childhood health and socioeconomic status was included, the odds for the 'unhealthy' cluster increased to 1.95 (95% CI: 1.10–3.45), indicating a stronger combined effect."*

Comments from Reviewer #2

Objectives

What do the authors want to emphasize in this study? From the Abstract to the Methods section, it is the relationship between health and socioeconomic status in childhood and cognitive function in later life. However, the Discussion section is mainly devoted to discussing other secondary outcomes. The discussion seemed to be geared toward the pre-determined conclusion that "improving education is important."

Authors' response

Thank you for your valuable observation. In response to your feedback, we have entirely rewritten the Discussion section to focus specifically on the primary study objectives and findings, ensuring a clear alignment with the relationship between childhood health, childhood SES, and risk of dementia in later life. We hope this revision adequately addresses your concerns regarding emphasis and enhances the clarity and relevance of the discussion.

Comments from Reviewer #2

Statistically, there is also the problem of multiple testing. It is recommended that the authors revisit their research design, including statistical analysis, focusing on what they wish to demonstrate.

<u>Authors' response</u>

Thank you for your valuable comment. In response, we have carefully reviewed and refined our study objectives and statistical analysis plan to pre-specify our primary hypotheses. This approach enables us to focus the interpretation on key tests of interest and mitigates concerns related to multiple testing. We have also clarified this strategy in the Methods section of the revised manuscript (Methods, Statistical Analysis, page 9, line 22 – page 10, line 22):

"Statistical Analysis

Most studies examining the influence of childhood conditions on dementia in later life have used retrospectively collected childhood conditions as the essential exposure 25 26. However, the use of such retrospective information gives rise to the potential problem of recall bias, especially among respondents with dementia risk. To address this bias, our study used LCA to objectively group childhood health and SES, reducing reliance on self-reports (see online supplemental figure S1).

Firstly, LCA was performed on childhood health and SES variables separately to identify clusters. The number of distinct clusters identified and chosen for final analyses was dependent on various criteria: a lower Akaike's Information Criteria (AIC) and Bayesian Information Criteria (BIC) with each class added to the model, an entropy value of at least 0.8 (indicating strong model accuracy and reliability) and at least 5% of the respondents within each class 27. Secondly, the frequency and percentage of each trait within the identified clusters were calculated. Covariates were reported by subgroup, with categorical variables presented as frequencies and percentages, and continuous variables as means and standard deviations. We performed bivariate analysis using the Chi-squared test for

categorical variables and the Kruskal-Wallis test for continuous variables. Then, multivariate ordinal logistic regression analyses were used to estimate the associations between childhood health, childhood SES, and their interaction with dementia risk within the identified latent classes. Three models were applied: Model 1 adjusted for age and sex; Model 2 further adjusted for SES and lifestyle factors i.e. employment, marital status, education level, wealth quintile, and social activities; and Model 3 additionally added health behaviours and health status i.e. smoking status, number of chronic diseases, and depression scores. We further performed analyses including both childhood health and SES in one model. Finally, we included the interaction between those two variables to understand if the effect of childhood health on high risk of dementia depends on the level of childhood SES and vice versa."

VERSION 2 - REVIEW

**Reviewer**	**1**
**Name**	**Bodryzlova, Yuliya**
**Affiliation**	**Institut National de Psychiatrie Légale Philippe-Pinel, Montreal, Canada**
**Date**	**02-Jul-2025**
**COI**	

Association of Childhood Health and Socioeconomic Status with Dementia Risk in Older Age: A Cross-Sectional Study Using the Indonesia Family Life Survey 2014–2015

Dear authors, editor,

Thank you for the opportunity to review this important manuscript. I find that the topic addressed and overall approach may have an important influence on policy and practice locally and globally.

While the manuscript has been thoroughly revised, there remain some important methodological concerns, which, I hope, are relatively easy to address.

1. You excluded missing values on exposure. It might have caused a selection bias. Could you use multiple imputation by chained equations to impute the missing data? Please find the manual (1).

2. You have a prevalence of dementia of about 13% in a population of 50 years old and over. It seems that your cut-off points were not validated in your target population. If there were, mention it in the methods section and discuss this high prevalence of dementia in the discussion section. If your cut-off points were not validated in your target population, consult the local experts to find cut-off points reflecting your reality.

3. Report the correlation between childhood poverty and childhood health.

4. Report the interaction according to recent guidelines: (2, 3), both additive and multiplicative.

5. Mention that the inclusion of depression makes the association between childhood health and dementia clinically insignificant.

6. For the sake of reproducibility and transparency, could you consider placing your code in a free depository?

References

1. van Buuren S, Groothuis-Oudshoorn K. mice: Multivariate Imputation by Chained Equations in R. Journal of Statistical Software. 2011;45(3):1-67.

2. VanderWeele TJ, Knol MJ. A Tutorial on Interaction. Epidemiol Methods. 2014;3(1):33-72.

3. Long JA. Interactions: Comprehensive, User-Friendly Toolkit for Probing Interactions. R package version 1.2.0, . CRAN; 2024.

Reviewer **2**

Name **Osaki, Tohmi**

Affiliation **Kobe Gakuin University Faculty of Rehabilitation, Department of Occupational Therapy**

Date **16-Jul-2025**

COI

The authors responded appropriately to my comments.

VERSION 2 - AUTHOR RESPONSE

REVIEWER 1 COMMENTS

Comments from Reviewer #1

1. You excluded missing values on exposure. It might have caused a selection bias. Could you use multiple imputation by chained equations to impute the missing data? Please find the manual (1).

1. van Buuren S, Groothuis-Oudshoorn K. mice: Multivariate Imputation by Chained Equations in R. Journal of Statistical Software. 2011;45(3):1-67.

Authors' response

Thank you for this valuable comment. We have addressed missing values across both exposure and covariate variables using multiple imputation by chained equations (MICE). We used the Stata-based approach described by:

White IR, Royston P, Wood AM. Multiple imputation using chained equations: Issues and guidance for practice. Stat Med. 2011;30(4):377–399. doi:10.1002/sim.4067.

In Methods, Sensitivity Analysis, page 10, line 25 – page 11, line 3, we now state:

"To assess the robustness of the main findings, we conducted a sensitivity analysis using multiple imputation using chained equations (MICE) to address missing data, following White and colleagues 30. The multivariate ordinal logistic regression models were re-estimated using the imputed data and compared with the original results to examine whether the observed associations remained consistent."

Corresponding results are reported in the Results section (page 17, lines 16–20):

"The sensitivity analysis using imputed data yielded results consistent with the main findings, reaffirming the associations between childhood health, childhood SES, and dementia risk in later life (see online supplemental tables S8 and S9). These findings reinforce the robustness of the original results and suggest that missing data did not substantially affect the observed associations."

Comments from Reviewer #1

2.1. You have a prevalence of dementia of about 13% in a population of 50 years old and over. It seems that your cut-off points were not validated in your target population. If there were, mention it in the methods section …

Authors' response

Thank you for this helpful comment. We acknowledge that the TICS-based cut-off points used in our study have not yet been formally validated in the Indonesian population. In our analysis, we adopted the categorisation developed by Langa et al. for the Health and Retirement Study (HRS) and its international sister studies, which are widely used in population-based surveys of individuals aged 50 and over, including the IFLS. Furthermore, a recently published study using IFLS data with the same target population (aged 50 and above) also employed the same TICS-based categorisation to assess cognitive outcomes: Sujarwoto S, Yumarni T, Holipah H, et al. Can socioeconomic disadvantage explain cognitive differences in later life? Insights from the Indonesia family life survey. Front Public Health 2025;13:1563543. doi: 10.3389/fpubh.2025.1563543.

While this supports the application of these thresholds in the Indonesian context, we recognise that they have not undergone formal local validation. To address this, we have added the following statement to the Methods section (Dementia Measures, page 8, line 25 – page 9, line 8):

"Following the classification approach developed by Langa et al. for the HRS and its international sister studies 22, participants were categorised into three groups based on their total TICS-27 score: 12–27 points indicating normal cognitive function, 7–11 points representing cognitively impaired but not demented (CIND), and 0–6 points signifying a high risk of dementia. These cut-offs have been used in prior studies and shown to have acceptable sensitivity and specificity in distinguishing levels of cognitive function in population-based settings where clinical diagnosis is not available 22 23. While a recent study using IFLS data

applied the same categorisation to assess cognitive outcomes 24, these thresholds lack formal validation in Indonesia, so prevalence estimates should be interpreted cautiously."

Additionally, we included this limitation in the Discussion (Strengths and Limitations, page 22, line 24 – page 23, line 4):

"However, several limitations should be acknowledged. First, dementia risk was inferred from a brief cognitive test (TICS) rather than clinical diagnosis. While this approach is commonly used in large-scale population studies, the TICS-based cut-off points have not been formally validated in the Indonesian context. We recommend that future studies involve clinical experts and undertake empirical validation to establish context-specific criteria. Incorporating clinical diagnostic confirmation would further enhance the reliability and validity of future research."

Comments from Reviewer #1

2.2. … and discuss this high prevalence of dementia in the discussion section. If your cut-off points were not validated in your target population, consult the local experts to find cut-off points reflecting your reality.

Authors' response

Thank you for your comment. We have included the discussion of the high prevalence of dementia in this study in the Discussion section (Discussion, page 18, lines 1– 21):

"Our findings showed that 15.67% of the sample were at high risk of dementia, which is notably lower than the 27.9% prevalence reported in another cross-sectional study by Farina et al. conducted in Indonesia 31. This discrepancy may be explained by differences in the age groups studied. While their study focused exclusively on individuals aged 65 and above, our study included participants aged 50 and over. Given that dementia risk increases with age 32, the older sample in their study likely contributed to the higher prevalence observed.

Nonetheless, the prevalence in our sample remains relatively high compared to similar studies in high-income countries. For instance, a study using the English Longitudinal Study of Ageing (ELSA) reported a dementia prevalence of 9.7% among adults aged 70 and over 33, while the US Health and Retirement Study (HRS) found a prevalence of 11.2% in a similar age group 33. A study in Sweden estimated a dementia prevalence of 12.5% among adults aged 65 and older 34. The higher prevalence in our Indonesian sample compared to these high-income countries likely reflects the cumulative impact of socioeconomic and health-related disadvantages prevalent in LMICs, such as Indonesia 31. Limited access to formal education, higher rates of childhood malnutrition, inadequate preventive healthcare, and increased exposure to infectious and non-communicable diseases may contribute to an earlier onset and elevated risk of dementia 35. These factors, compounded across the life course, create a higher burden of dementia risk in Indonesia compared to high-income settings with better healthcare infrastructure and socioeconomic conditions."

While we fully recognise the value of consulting local clinical experts, to the best of our knowledge, no formally validated TICS cut-off points currently exist for the Indonesian population. As this study makes use of secondary, standardised survey data and aims to ensure comparability with international ageing studies, we applied the harmonised thresholds developed by Langa et al., which are commonly used in similar contexts. We agree that the development of locally validated thresholds would be highly beneficial, and we hope future research will address this important gap. To further address your suggestion to involve local experts, we have added the following to the Conclusion section (page 23, line 20–22):

"In addition, the development of locally validated cognitive assessment tools is essential to improve diagnostic accuracy and ensure culturally appropriate measurement of dementia risk in diverse settings."

<u>Comments from Reviewer #1</u>

3. Report the correlation between childhood poverty and childhood health.

Authors' response

Thank you for your helpful suggestion. We have added the correlation between childhood SES and childhood health in the Results section (page 16, line 14 – page 17, line 4):

"To investigate the relationship between childhood health and childhood SES, we conducted a chi-squared test and computed a tetrachoric correlation (see online supplemental table S6). A higher proportion of individuals classified in the 'poor SES' group reported 'unhealthy' childhood (9.43%) compared to those in the 'non-poor SES' group (6.69%). The chi-squared test confirmed a statistically significant association between them (χ^2=6.09, p=0.014. In addition, the tetrachoric correlation (ρ=0.094, SE=0.037, p=0.013) indicated a modest but significant positive relationship, suggesting that 'poor SES' childhood was associated with an increased likelihood of experiencing 'unhealthy' childhood health."

Comments from Reviewer #1

4. Report the interaction according to recent guidelines: (2, 3), both additive and multiplicative.

2. VanderWeele TJ, Knol MJ. A Tutorial on Interaction. Epidemiol Methods. 2014;3(1):33-72.

3. Long JA. Interactions: Comprehensive, User-Friendly Toolkit for Probing Interactions. R package version 1.2.0, . CRAN; 2024.

Authors' response

Thank you for your valuable comment and the recommended references. We have re-conducted the interaction analysis following the tutorial by VanderWeele and Knol (2014). This has been clarified in the Methods section (Statistical Analysis, page 10, lines 21–23):

"Finally, we assessed multiplicative and additive interactions between childhood health and childhood SES on dementia risk, following guidelines for interaction analysis 29"

We have reported the results in the Results section (page 17, lines 4–15):

"We further assessed the interaction between childhood health and SES on dementia risk in table 4. In logistic regression models fully adjusted for age, sex, employment, marital status, education, wealth, social capital, smoking, chronic diseases, and depression, poor SES was associated with increased dementia risk without interaction (p=0.048), but this effect was not significant with interaction (p=0.052). Unhealthy childhood showed non-significant effects both without interaction (p=0.061) and with interaction (p=0.496). The multiplicative interaction was not significant (OR=0.88, 95% CI: 0.30–2.57, p=0.809), nor were additive interactions, measured by relative excess risk due to interaction (RERI=-0.06, p=0.935), attributable proportion (AP=-0.04, p=0.935), and synergy index (S=0.93, p=0.259). These results suggest that the interaction between unhealthy childhood and poor SES does not significantly amplify the dementia risk beyond their individual effects."

This is also discussed in the Discussion section (Interaction Between Childhood Health and SES, page 21, line 26 – page 22, line 6):

"While both poor childhood SES and unhealthy childhood health were individually associated with increased dementia risk, our findings did not reveal significant multiplicative or additive interactions between them. This suggests that their effects on dementia risk are independent rather than synergistic. The absence of significant interaction may reflect the multifactorial nature of dementia development and the influence of mediating factors across the life course, such as adult SES, health behaviours, and access to care 1."

Comments from Reviewer #1

5. Mention that the inclusion of depression makes the association between childhood health and dementia clinically insignificant.

Authors' response

Thank you for your thoughtful feedback. We have addressed this point in both the Results and Discussion sections. In the Results (page 14, lines 5–8), we now state:

"In the fully adjusted model including depression, the association between childhood health and dementia risk was no longer statistically significant, suggesting that depression may mediate this relationship."

We have also included the following in the Discussion (Later-Life Covariates, page 21, lines 16–19):

"Furthermore, the inclusion of depression in the model attenuated the association between childhood health and dementia risk to non-significance. This finding suggests that later-life depression may partially mediate the long-term impact of childhood health on dementia risk, consistent with findings from other studies 52 53"

Comments from Reviewer #1

6. For the sake of reproducibility and transparency, could you consider placing your code in a free depository?

Authors' response

Thank you for your helpful suggestion. We have uploaded the Stata syntax to Zenodo and included the DOI in the manuscript (page 31, lines 1–4):

"Data availability statement: The data used in this study are publicly available from the RAND's Labor and Population website: https://www.rand.org/labor/FLS/IFLS.html. The Stata syntax used for data preparation and analysis is openly accessible via Zenodo at https://doi.org/10.5281/zenodo.16179662."
